# Properties, Preliminary Risk Evaluation and Potential Valorization of *Miscanthus × giganteus* Biomass Ash as a Soil Amendment

**DOI:** 10.3390/toxics14070541

**Published:** 2026-06-23

**Authors:** Abdulmannan Rouhani, Karim Suhail Al Souki, Batoul Hamade, Ghazwa Basma, Petr Ryšánek, Valentina Pidlisnyuk

**Affiliations:** 1 Department of Environment, Faculty of Environment, Jan Evangelista Purkyně University in Ústí nad Labem, Pasteurova 15, 400 96 Ústí nad Labem, Czech Republic; abdulmannan.rouhani@ujep.cz (A.R.); batoulhamade31@gmail.com (B.H.); ghazwabasma99@gmail.com (G.B.); 2 Department of Environmental Chemistry and Technology, Faculty of Environment, Jan Evangelista Purkyně University in Ústí nad Labem, Pasteurova 15, 400 96 Ústí nad Labem, Czech Republic; valentina.pidlisnyuk@ujep.cz; 3 Centre for Nanomaterials and Biotechnology, Faculty of Science, Jan Evangelista Purkyně University in Ústí nad Labem, Pasteurova 15, 400 96 Ústí nad Labem, Czech Republic

**Keywords:** *Miscanthus × giganteus* biomass ash, soil amendment, nutrient recycling, potential toxic elements, polycyclic aromatic hydrocarbons, by-products of biomass combustion

## Abstract

The agricultural and environmental application of *Miscanthus × giganteus* biomass ash (MBA) as a soil amendment requires a thorough assessment of its properties, nutrient potential, and associated risks. This study characterizes the elemental composition, pH, cation exchange capacity (CEC), and polycyclic aromatic hydrocarbons (PAHs) content of MBA in comparison with other common biomass ashes (crops, wood, and sewage sludge) referred to the international regulatory standards. The ash exhibits a strong alkaline pH (11.03), suggesting potential to improve soil pH in acid soils, but requires careful controlled application to prevent excessive alkalization. The main nutrients detected include K (5.54%), Ca (2.07%), Mg (0.37%), and P (0.86%), indicating its potential as a soil amendment, though long-term use may cause nutrient imbalances. Micronutrients such as Zn (240.67 mg·kg^−1^), Mn (297 mg·kg^−1^), and Cu (33.5 mg·kg^−1^) are found in concentrations suitable for agricultural use, while potentially toxic elements (PTEs), including Cd, Cr, Ni, and Pb, are below detection limits, thereby reducing the risk of pollution. As (8.3 mg·kg^−1^) and ΣPAHs (1.63 mg·kg^−1^) remain within safety thresholds, suggesting a low environmental toxicity of MBA. The low Na content (0.12%) indicates a minimal risk of salinity accumulation, distinguishing MBA from high-sodium biomass ashes. Soil alkalization, disruptions in nutrient balance, and element leaching are risks to be considered. Despite these concerns, its composition is in agreement with established safety guidelines, supporting its feasibility for valorization as a sustainable soil amendment and remediation material. To maximize agronomic benefits and mitigate environmental risks, it is important to utilize the ash, considering site conditions and carry out regular monitoring of the soil.

## 1. Introduction

*Miscanthus × giganteus (M × g)* is widely recognized as a highly effective second-generation biofuel crop, offering an eco-friendly substitute for conventional energy sources [1,2]. This crop is particularly well-adapted to low-temperature environments, exhibiting a rapid growth cycle, high biomass yield, and substantial carbon sequestration potential [3,4]. By the third year of cultivation, *M × g* plantations reach their peak biomass productivity, characterized by high cellulose content. Once established, the crop can be harvested annually for about 25–30 years, demonstrating long-term viability as a reliable biofuel source [5,6]. The energy capacity of *M × g* biomass is comparable to that of wood, showing its potential as an alternative energy resource [7]. In addition, its harvested biomass typically possesses low moisture content, a critical factor for increasing energy yield. Consequently, *M × g* boasts a high net energy content compared to other energy crops [8].

Biomass combustion for power generation is a practical and reliable approach to achieving energy neutrality objectives [9]. However, the combustion of biomass generates by-products, such as ash, and due to the growing use of alternative energy, the volume of ash produced by combustion has been rising steadily [10]. It should be noted that herbaceous biomass ash contains a higher proportion of alkali metals compared with wood or coal when incinerated. This can give rise to a number of significant operational issues, including fouling and slagging, as well as environmental challenges [11]. Accordingly, beyond its role as an energy crop, *M × g* can be deployed in phytomanagement strategies on contaminated land, where it reduces contamination risks while simultaneously producing harvestable biomass for bioenergy [2]. Following harvest, this biomass is typically combusted for energy generation, resulting in the production of ash [12]. However, ash quality is strongly influenced by biomass origin, raising potential concerns when feedstocks are derived from contaminated sites. To date, ash-related research has largely focused on wood and conventional biomass sources, whereas data on crop-derived ashes, particularly those originating from remediation systems, remain limited [13]. Consequently, the properties and potential environmental risks associated with *M × g*-derived biomass ash from phytomanagement systems remain poorly characterized, especially when both potentially toxic elements (PTEs) and polycyclic aromatic hydrocarbons (PAHs) must be jointly considered to assess safe soil application.

The management of energy crops’ biomass ash has attracted considerable interest because of its broad implications in clean energy technology. The disposal of ash presents several challenges, as it often leads to environmental challenges, including leachate contamination and land use limitations [14]. From that prospective the potential valorization of biomass ash is increasingly gaining traction, creating feasible solutions instead of landfill disposal. The valorization actions included: ash incorporation in silicon-based materials [15], pavement materials [16], zeolite synthesis and catalysis [17,18], ceramics and concrete production [19], and utilization as soil amendments and fertilizers [20,21]. The feasibility of these applications is based on the properties of the ash, i.e., chemical composition, mineralogy, and physical characteristics [22]. Among different valorization pathways, one of the most environmentally sustainable strategies is its return to the soil as a soil amendment [10]. This material provides a substantial amount of essential plant nutrients, including Ca, K, Mg, and P, which are relatively soluble and bioavailable to plants [12,23]. Numerous studies have demonstrated that the incorporation of biomass ash into agricultural soils significantly improved soil properties, supported the growth of plants, and increased crop productivity. This practice also supported the natural mineral cycle and decreased reliance on synthetic fertilizers, thereby contributing to the long-term stability of agricultural production [24,25]. Additionally, biomass ash being used as an amendment promoted carbon sequestration by enhancing the humification of organic material, thereby increasing carbon stability in soils [22,26].

The utilization of biomass ash in agricultural and environmental sectors has the potential to transform it from a waste product into a beneficial material, effectively minimizing landfill disposal [9,27]. However, the highly alkaline nature of biomass ash and the presence of PTEs required careful management of its application. Ash may also contain some persistent organic pollutants, including polycyclic aromatic hydrocarbons (PAHs), which result from incomplete combustion and flue gas reactions [28,29]. Based on this, we expected ΣPAHs to be low when combustion was largely complete. These risks highlight the importance of comprehensive essential safety assessments to minimize environmental risks after valorization of ash. Sustainable ash application requires establishing and implementing effective management strategies for the various ash fractions generated during biomass combustion [30,31].

A critical aspect of the sustainable management of biomass ash from large-scale combustion facilities involves detailed characterization of its types and properties. These attributes provide an essential basis for creating standards that determine ash’s applicability in environmental and industrial settings [32]. Given the global abundance of biomass ash, planned research on the valorization of biomass ash has ensured advanced sustainability and effective resource management. *M × g* biomass cultivated under phytomanagement on PTE-contaminated soil can produce an ash that meets key guideline values for land application despite the contaminated origin of the feedstock. We further hypothesize that regulated PTEs (e.g., Cd, Cr, Ni, and Pb) remain low or below detection limits in the ash, while certain elements (e.g., Zn) may still be limiting depending on the regulatory framework. Therefore, this study aims to conduct an in-depth analysis of the properties, nutrient value, and preliminary environmental risk profile of *M × g* biomass ash (MBA) to examine its viability for agricultural and environmental applications as a valorized product after combustion of *M × g* biomass. Specifically, this study quantifies PTEs and ΣPAHs in MBA and compares them to guideline values, as well as key physicochemical parameters (pH, CEC, and nutrient content) relevant to controlled soil applications. By analyzing its elemental composition, pH, CEC, and PAH content, and comparing the results with other biomass ashes and international safety guidelines, this research provides a thorough assessment of its potential as a soil amendment. While biomass ash is widely recognized for increasing soil nutrient availability and functioning as a liming agent, concerns regarding soil alkalization, PTEs leaching, microbial shifts, and disturbances in nutrient cycling necessitate careful assessment. Understanding the influence of these factors is essential for developing a framework for the valorization of the *M × g* BA, while ensuring environmental safety.

## 2. Materials and Methods

### 2.1. Ash Background

**Figure 1:**
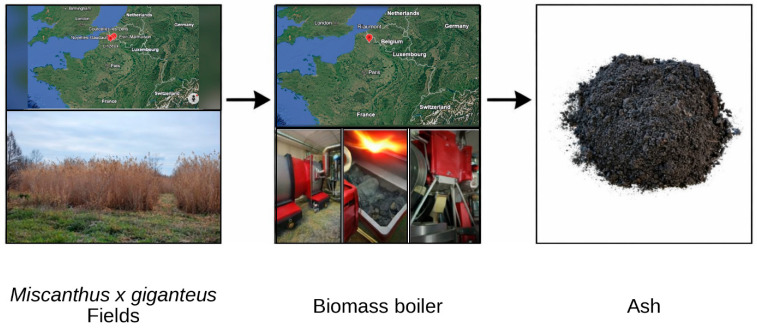
Locations of cultivated *M × g*, power plant, and byproduct in the form of ash.

The tested biomass ash was sourced from a power station near Lille, France, where *M × g* is utilized as a renewable energy feedstock (Figure 1). The biomass was cultivated on PTE-contaminated land at a site significantly impacted by atmospheric dust emissions from the former Metaleurop Nord smelter throughout the 20th century. This location represents a long-term contaminated site, with soil heavily polluted by Cd, Pb, and Zn, along with elevated concentrations of Mn, Cr, Cu, and Sr at levels 20–50 times above regional background values [33]. Owing to the complicated characteristics of this contaminated mega-site, characterized by extensive polluted soils and associated human and environmental risks, phytomanagement with *M × g* was proposed as a sustainable remediation strategy. This approach enables soil stabilization while minimizing reclamation costs and increasing biomass valorization. The harvested biomass was subsequently repurposed for alternative energy production, contributing to ecological well-being and financial viability [34]. The sampled ash was collected from the bottom ash fraction.

### 2.2. Ash Characterization

The MBA samples were air-dried at room temperature, gently crushed, and passed through a <2 mm sieve. The resulting WBA fraction was then thoroughly mixed to achieve homogeneity prior to subsampling. Samples for each analysis were obtained from the combined bulk material. The pH of WBA was determined with a glass electrode in a 1:10 ash-to-deionized water mixture after 1 h of shaking, using a benchtop multiparameter meter (inoLab Multi 9430 IDS, WTW, Weilheim, Germany). Cation exchange capacity (CEC) was measured using the ammonium acetate method, involving percolation with ammonium acetate followed by extraction with sodium chloride (NaCl) (ISO/TS 22171, 2023 [35]). The ammonium ions (NH_4_^+^) were then quantified by spectrophotometry (SpectraMax^®^ 190, Molecular Devices, San Jose, CA, USA) according to ISO 7150/1 (1984) [36]. Proximate analysis, assessing water content, volatiles, ash content, and fixed carbon, was completed using the thermogravimetric multi-analyzer system (TGA version 1.4.3.6 by Leco, West, Germany), in accordance with ASTM D1762-84 (2021) [37]. Elemental composition of ash consisting of C, H, N, S, and O was measured using Flash 2000 (Thermo Scientific, USA).

The chemical composition of ash was completed with a wave dispersive X-ray fluorescence spectrometer (WDXRF), Rigaku Primus IV model (Rigaku, Tokyo, Japan) with the SQX software, which enables the measurement of elemental concentrations ranging from F to U, with detection limits ranging from ppm to 100% [38]. Quantification relied on fundamental parameter calibration with matrix correction. Method performance was checked using certified reference materials and replicate measurements (RSD < 5%). Detection limits for trace elements ranged between 1 and 10 mg·kg^−1^, while limits of quantification (LOQs) were calculated as ten times the standard deviation of blank measurements. The quantification of PAHs was done following Al Souki et al. [4], using a gas chromatography (GC) (7890B, Agilent Technologies, USA) coupled to a triple quadrupole mass spectrometer (MS) (7000D, Agilent Technologies, USA). A split-less liner double-taper UI (Agilent, USA) was used together with a DB-EUPAH chromatographic column (20 m × 0.18 mm, 0.14 µm, Agilent, USA). Helium (5.5) was used as the carrier gas and nitrogen (6.0) as the collision gas. Sixteen EPA priority PAHs of focus were measured [4]. Quantification was performed using multi-point calibration with internal standards (r^2^ ≥ 0.995). Each analytical batch included blanks, matrix spikes, and duplicate samples. Detection limits for individual PAHs were 0.1–0.5 μg·kg^−1^, and LOQs were calculated as ten times the standard deviation of replicate low-level spikes.

### 2.3. Statistical Analysis

A statistical analysis was performed in Microsoft Excel. Built-in statistical functions, such as the arithmetic mean and standard deviation, were employed. The results obtained from the experiment were compared with relevant reference values from the literature and applicable guideline limits where available.

## 3. Results and Discussion

The characterization of MBA provides valuable information about its chemical composition, potential applications, and possible threats within agricultural and ecological settings. This section examines its chemical properties, elemental composition, and pollutant concentrations, comparing them with biomass ashes reported in the literature. The discussion evaluates the suitability of MBA as a soil amendment, considering its concentrations of essential macro- and micronutrients, alkalinity, and PTEs concentrations. Additionally, associated risks, including soil alkalization, imbalances in nutrients, and the leaching of PTEs and PAHs, are assessed against international legal requirements. Using a combination of comparative data and existing guidelines, this section thoroughly assesses the practicality and challenges of applying MBA in sustainable land management.

### 3.1. MBA Characteristics

Table 1 presents the cation exchange capacity (CEC), water content (WC), volatile matter (VM), ash content, and fixed carbon (FC) of MBA, compared with data on biomass’s ashes presented in the literature.

The CEC of MBA in this study was 1.77 ± 0.36 cmol kg^−1^, significantly lower than wood ash (59.8 cmol kg^−1^), rice husk ash (40.0 cmol kg^−1^), rice mill ash (17.64 cmol kg^−1^) and sago bark ash (13.13 cmol kg^−1^), highlighting further its limited role in improving soil nutrient retention. This suggests that MBA is one of the lowest CEC values among the others. Its application may benefit nutrient-depleted soils, but is most effective when combined with organic amendments or high CEC materials to improve long-term soil nutrient retention. For environmental purposes, the low CEC of MBA suggests its ability to stabilize contaminants and improve soil structure might be limited for soil remediation and PTEs immobilization. Additionally, the low nutrient-retention capacity contributes to an elevated risk of nutrient loss through leaching, particularly in sandy soils or high-rainfall environments where essential nutrients could be rapidly lost [45]. Unlike wood ash and rice husk ash, which promote improvements in soil texture and cation retention [41,46], the impact of MBA may be less significant on long-term soil fertility. Furthermore, in soils with low buffering capacity, their high alkalinity could exacerbate nutrient imbalances, affecting nutrient uptake by plants.

The WC of MBA in this study was 0.47 ± 0.02%. Several studies showed that direct comparison with WC moisture content and water holding capacity due to the variability in experimental procedures. However, the low WC observed is consistent with expectations for biomass ash, indicating minimal residual moisture following combustion. This characteristic is relevant for storage and application, since moisture content influences ash dispersibility, reactivity, and its engagement with soil and environmental systems [47,48]. The VM content of MBA was 3.92 ± 0.44%, substantially lower than that of high-carbon wood ash (19.63%) and general wood ash (20%) but higher than mixed biomass ash (1.17%). The lower VM content suggests a more complete combustion process, reducing the occurrence of unburned carbonaceous compounds. As a result, MBA has a reduced potential for post-combustion emissions and a more stable inorganic composition [49], which might determine its interaction potential and suitability for agricultural and environmental applications.

The ash content of MBA in this study was 94.23 ± 0.27%, significantly higher than that of high-carbon wood ash (26.15%) and general wood ash (70.4%) but slightly lower than mixed biomass ash (98.67%) and rice husk ash (96.0%). The high ash content indicates a low proportion of organic combustible material, suggesting a high degree of mineralization during incineration. Compared to wood-derived ashes, which retain a substantial fraction of unburned carbon, MBA closely resembles rice husk ash and mixed biomass ash, which mainly have higher mineral content due to the combustion of lignocellulosic feedstocks with inherently greater inorganic fractions. The high ash content in MBA suggests its suitability for uses that need stable, mineral-rich material, such as soil amendments or materials for construction purposes.

The FC content of MBA in this study was 1.85 ± 0.18%, significantly lower than that reported for wood ash (9.6%) but higher than in mixed biomass ash (0.11%). The relatively low content of FC indicates that *M × g* biomass undergoes efficient combustion, leaving minimal unburned carbon in the ash residue. Compared to wood ash, which retains a higher proportion of FC due to incomplete combustion or the presence of recalcitrant organic matter, MBA exhibits a composition closer to that of mixed biomass ash. The low FC content implies a reduced potential for post-combustion oxidation reactions, increasing its stability for agricultural and environmental applications.

Table 2 presents the elemental composition of MBA, including carbon (C), nitrogen (N), hydrogen (H), sulfur (S), and oxygen (O), in comparison with biomass ashes reported in the literature.

The total C content of MBA in this study was 5.34%, higher than values reported for wood ash (3.25%), *M × g* ash from Croatia (3.39%), mixed biomass ash (2.99%), sorghum ash (3.42%), and Jerusalem artichoke ash (3.40%). However, it is significantly lower than that of wood pellet ash (69.2%), buckwheat husk ash (29.53%), wheat straw fly ash (20.9%), and bagasse ash (10.4%). This suggests that MBA retains more carbon due to differences in combustion temperature and feedstock composition. The total carbon content of MBA suggests potential utilizations in agricultural and environmental management, notably as a soil amendment. Carbon in ash can affect soil properties by improving structure, water-holding capacity, and microbial activity [62,63]. Additionally, the presence of C can affect the ash’s ability to adsorb pollutants and nutrients [64], thereby making it potentially useful for soil remediation and as a stabilizing agent for PTEs. Although MBA has a lower total C content than biochar or other high-carbon biomass ashes, it still provides a carbon input that may improve organic matter levels in disturbed soils. Many studies have proved that soil amendment with high doses of biomass ash increased the content of C in soil [65,66,67]. When applied environmentally, total C can contribute to the ash’s reactivity and could influence its contribution to carbon sequestration [68], while its performance would be lower than that of biochar or other carbon-rich materials.

The total N content of MBA in this study was 0.02%, higher than that reported for *M × g* ash from Croatia (0.005%) and mixed biomass ash (0.001%) but lower than wood pellet ash (0.15%), sorghum ash (0.50%), Jerusalem artichoke ash (0.40%), and buck-wheat husk ash (0.66%). The N content in MBA also exceeded the detection limit (LOD), its concentration remains relatively low, indicating some nitrogen retention post-combustion. The relatively low N content is consistent with expectations, as most N volatilizes during combustion, so that only a negligible amount remains in biomass ash. Consequently, MBA is unlikely to function as a significant N fertilizer, indicating that depending exclusively on this ash for soil fertility enhancement could bring about N depletion, necessitating supplementation with additional fertilizers or organic amendments. Many studies reported low N content in biomass ash-amended soils [69,70,71]. Even with minimal N, it may promote microbial processes and nutrient dynamics in amended soils.

The H content of MBA in this study was 0.14%, significantly lower than the values reported for wood pellet ash (1.4%) and wheat straw fly ash (0.62%). No comparable data were available for other biomass ashes. The relatively limited H content indicates that most hydrogenous compounds, primarily resulting from organic matter and water, were volatilized during combustion. MBA has undergone a more complete decomposition of hydrogen-containing organic compounds, producing a highly mineralized final product. The low H content indicates a minimal presence of volatile compounds and hydrocarbons, enhancing the stability of MBA as a soil amendment. Unlike high-hydrogen biomass ashes, which may retain unburned organic material, the low H level suggests that MBA is more chemically stable when incorporated into soils, decreasing the potential for continued decomposition or reactive interactions in agricultural and environmental applications.

The S content of MBA in this study was 0.19%, higher than that reported for wood pellet ash (0.10%), sewage sludge ash (0.036%), and bagasse ash (0.05%) but lower than wood ash (1.72%), mixed biomass ash (0.47%), rice straw and husk ash (1.8%), coal fly ash (0.5%), and wheat straw fly ash (2.73%). The relatively low content of S shows that *M × g* biomass contains fewer S-containing compounds than biomass sources with higher S levels, where elevated S may result from S-rich feed stocks or combustion conditions that promote S retention in the ash matrix. S is an essential plant nutrient, which is vital for protein production and enzymatic activity [72,73]. While the S content in MBA is lower than in some biomass ashes, it remains a source of S availability in soils, particularly in S-deficient regions. Moreover, the relatively low S content minimizes the risk of excessive soil acidification, making MBA a suitable amendment for sustaining soil fertility without significant pH reduction.

The O content of MBA in this study was 94.41%, significantly higher than that of wood pellet ash (24.9%), rice straw and husk ash (46.4%), rice husk ash (26.5%), and wheat straw fly ash (6.83%). No O content was reported for wood ash, *M × g* ash from Croatia, mixed biomass ash, or other biomass sources in the available studies. The exceptionally high O content in MBA indicates that it consists predominantly of oxides, likely including silica (SiO_2_), calcium oxide (CaO), magnesium oxide (MgO), and other metal oxides, reflecting an advanced state of mineralization during incineration. The high O level highlights that *M × g* undergoes more complete oxidation, reducing the proportion of residual C and VM. Such a composition is highly suitable for use as a soil amendment and for use in environmental uses requiring alkaline or mineral-rich materials. Its oxide-rich nature likely contributes to the regulation of soil pH, providing liming effects similar to high-Ca biomass ashes. In agricultural applications, the abundance of oxides may improve soil structure, availability of nutrients, and microbial dynamics by improving CEC [74,75,76]. In addition, because of its mineral characteristics, MBA may be effective as a cementitious additive in construction materials, similar to high-silica biomass ashes such as rice husk ash [77].

Table 3 presents the chemical composition and pH of MBA in comparison with various biomass ashes reported in the literature, showing variations in elemental concentrations and alkalinity.

The pH of MBA in this study was 11.03 ± 0.04. Even though this value was lower than biomass fly ash from residual forest biomass (13.0), mixed biomass ash from agricultural and animal wastes (12.39), sorghum ash (12.0), and wood ash (12.7), MBA remains highly basic with strong liming potential, making it more suitable for agricultural applications where soil pH adjustment is required. The high pH confirms the strong alkalizing properties of MBA, which may substantially affect soil and environmental systems. Due to its alkalinity, MBA is well-suited as a liming agent for acidic soils, effectively neutralizing acidity, improving soil conditions and plant growth. Many studies exhibited that biomass application leads to a significant increase in soil pH, with the highest doses showing the strongest effect [20,24,25,26]. Moreover, Uwiringiyimana et al. [83] showed that biomass ash had a stronger liming effect than biochar. Nevertheless, the high pH and alkaline cation content of biomass ash may contribute to the rise of soil salinity when used as a fertilizer, which could result in environmental challenges [10]. According to several studies, over-application may lead to micronutrient deficiencies, particularly for elements such as Fe, Mn and P, which become less bioavailable [84,85]. In recent years, aerobic decomposition and the integration of biomass ash with organic fertilizers, including sludge, farmyard manure, and biochar, have been recognized as proven approaches to reduce these risks [86,87,88]. These methods regulate the release rate of mineral elements from ash, enhance soil physical and chemical properties, and support the growth and development of plants [89]. In environmental applications, MBA could be utilized for acid neutralization in wastewater treatment, mine reclamation, and the stabilization of acidic waste materials. Additionally, depending on soil properties, increased alkalinity may increase the availability and mobility of certain elements, including PTEs, posing environmental risks [90].

The Al content of MBA in this study was 0.12 ± 0.01%, higher than mixed biomass ash (0.05%) and *Miscanthus sinensis* ash (0.1%) but lower than biomass fly ash from residual forest biomass (2.81%), sewage sludge ash (0.89%), and wood ash (1.24%). This indicates that MBA contains moderate to low levels of Al, compared to other biomass ashes. The relatively lower Al content suggests that *M × g* biomass contains fewer aluminosilicate minerals, which commonly occur in larger concentrations in woody and mineral-rich feedstocks. However, its Al content remains higher than that of some agricultural biomass ashes, indicating that a measurable fraction of Al is retained post-combustion. The moderate Al content in MBA suggests that it can be used in agricultural use with limited risk of toxicity, particularly in acidic soils where excessive Al availability can restrict plant growth and soil organisms [91]. Unlike high-Al biomass ashes, such as wood ash and sewage sludge ash, MBA is unlikely to cause Al-induced soil acidity problems. However, given the high pH of MBA, most of the Al is likely in stable, insoluble forms, reducing the risk of leaching or bioavailability under normal use rates. MBA presents a reduced risk of Al-related soil contamination but should still be monitored in long-term applications to prevent accumulation tendency in soils with repeated use.

The Ca content of MBA in this study was 2.07 ± 0.13%, comparatively lower than most other biomass ashes. Biomass fly ash from residual forest biomass (23.9%) and wood ash (13.5%) contains significantly higher levels of Ca, improving their performance as liming agents. Similarly, mixed biomass ash (5.99%), *M × g* ash from Poland (4.65%), *Miscanthus sinensis* ash (3.0%), and sorghum ash (3.84%) all exhibit higher Ca content than the MBA analyzed in this study. Only sewage sludge ash (1.76%) has a slightly lower Ca concentration. The relatively low Ca content in MBA suggests it is less enriched in Ca-bearing compounds, such as calcium oxides (CaO) and carbonates (CaCO_3_), than other biomass ashes. Despite this, MBA can be utilized as a supplementary liming material in agriculture, though it is less effective than wood ash or other high-Ca ashes in neutralizing soil acidity. It may still aid in improving Ca availability and soil texture, especially in nutrient-deficient soils. Additionally, the presence of Ca in biomass ash can enhance CEC and increase nutrient retention [92,93]. In environmental remediation, Ca in ash can facilitate the stabilization of contaminants by forming insoluble precipitates, thereby supporting its use in soil remediation and PTEs immobilization strategies [94]. However, due to its lower Ca content, MBA may be less effective in such applications than high-Ca ashes, such as wood ash.

The Fe content of MBA in this study was 0.24 ± 0.32%, comparable to MBA from Poland (0.25%) and slightly higher than sorghum ash (0.23%) and mixed biomass ash (0.22%). However, it is significantly lower than biomass fly ash from residual forest biomass (2.68%) and wood ash (0.67%) but notably higher than sewage sludge ash (0.02%). The relatively low Fe content in MBA suggests a lower concentration of Fe oxides or ferric compounds, reducing its effectiveness as a Fe-rich soil amendment. In addition, it can still be a supplementary Fe source for crops, particularly in Fe-deficient soils, though its overall contribution to soil Fe enrichment would be minimal compared to high Fe biomass ashes. Fe is vital to plant metabolic processes, notably in chlorophyll formation and enzymatic functions [95]. Regarding environmental uses, Fe-containing ashes could be used for contaminant stabilization due to their capacity to bind PTEs and P [96]. While an MBA may contribute to such applications, its Fe content suggests a lower adsorption potential compared to Fe-rich ashes.

The K content of the MBA in this study was 5.54 ± 0.14%, relatively high compared to most biomass ashes. It exceeds the K content of wood ash (3.94%) and *Miscanthus sinensis* ash (2.0%), is comparable to mixed biomass ash (5.31%), and is slightly lower than biomass fly ash from residual forest biomass (6.13%). However, it remains significantly lower than sorghum ash (16.3%) and moderately lower than *M × g* ash from Poland (8.74%). The very low K content in sewage sludge ash (0.21%) indicates that MBA retains a substantially higher K fraction than waste-based ashes. While MBA exhibits a high K concentration compared to most biomass ashes, it does not reach the extreme levels found in sorghum ash. The relatively high retention of K in MBA suggests that its feedstock naturally accumulates K and that combustion conditions preserved a significant portion of K rather than promoting volatilization. Due to that, MBA offers a reliable source of K for soil amendments, notably in K-poor agricultural lands. Utilization of biomass ashes in soil was shown to increase the content of K by many studies [24,41,52]. It offers a stronger K contribution than wood ash, establishing it as a feasible replacement for conventional K fertilizers. The solubility of K in biomass ash assures that it is highly bioavailable to plants, promoting important physiological activities such as water uptake, enzyme activation, and resistance to abiotic stress [97,98]. Within ecological restoration practices, high-K ashes are useful for soil rehabilitation and revegetation projects, where their availability facilitates faster vegetation development in degraded soils. The application of MBA may also support nutrient dynamics within forest ecosystems, as K is a critical element in preserving ecosystem function [99]. However, K’s high solubility increases leaching risk, particularly in sandy or highly permeable soils, leading to nutrient depletion and limited long-term supply. Excessive K inputs can also disrupt soil nutrient balance, potentially antagonizing Ca and Mg uptake by plants [100]. Additionally, if leached into aquatic environments, elevated K concentrations can cause eutrophication, particularly when located adjacent to water systems [101].

The Mg content of MBA in this study was 0.37 ± 0.02%, which is moderate to low compared to other biomass ashes. It is lower than biomass fly ash from residual forest biomass (2.16%), wood ash (1.27%), sorghum ash (0.99%), and mixed biomass ash (0.86%) but higher than *Miscanthus sinensis* ash (0.12%) and slightly lower than sewage sludge ash (0.39%). This suggests that MBA retains moderate Mg levels, higher than some agricultural biomass ashes but significantly lower than those of wood-derived ashes, which often contain higher Mg concentrations due to greater mineral accumulation in woody feedstocks. Its moderate Mg concentration suggests that it can be a supplementary source of Mg in soil amendments, particularly in Mg-deficient soils. Mg is essential for the production of chlorophyll and enzyme activation in plants, and its presence in biomass ash can improve soil fertility [102,103]. While MBA provides some Mg, it is less effective than high-Mg amendments such as wood ash or dolomite lime. However, its Mg content can still assist soil structure stabilization and CEC, enhancing nutrient retention [104]. In environmental applications, Mg-containing ashes can be utilized for contaminant stabilization, particularly for PTEs immobilization in polluted soils [105].

The Na content of MBA in this study was 0.12 ± 0.01%, which is low compared to most other biomass ashes. It is significantly lower than biomass fly ash from residual forest biomass (2.18%), wood ash (1.05%), and mixed biomass ash (0.77%). It also contains less Na than sewage sludge ash (0.21%) but more than *Miscanthus sinensis* ash (0.07%). No data were available for *M × g* ash from Poland or sorghum ash. The relatively low Na content suggests minimal Na accumulation in the plant material prior to combustion. Due to its low Na concentration, MBA can be utilized in agricultural soils safely without contributing to salinity issues. Unlike high-Na ashes, such as biomass fly ash (2.18%) and wood ash (1.05%), which often cause higher soil electrical conductivity [106] and induce osmotic stress in plants [107], MBA has minimal impact on soil salinity, ensuring its suitability for use in salt-sensitive cropping systems. Although Na plays a limited role in plant nutrition, it can partially substitute for K in some metabolic functions. When applied in environmental management, the low Na content of MBA makes it a favorable option for soil remediation and stabilization, as excessive Na could otherwise degrade soil structure through increasing clay dispersion and reducing permeability [108]. The minimal Na concentration in MBA expands its use range in diverse agricultural and environmental contexts with low risk of salinity issues.

The P content of MBA in this study was 0.86 ± 0.08%, classifying it as moderate within the range of P concentrations reported in biomass ashes. While lower than high P ashes such as sewage sludge ash (3.56%) and *M × g* ash from Poland (2.48%), it is higher than biomass fly ash (0.49%) and *Miscanthus sinensis* ash (0.06%). It is also slightly lower than wood ash (1.0%), mixed biomass ash (1.47%), and sorghum ash (1.42%), though these differences are not extreme. Although its P level is lower than that of some biomass ashes, MBA can still be a supplementary P source in agriculture. P is vital for root development, flowering, and energy transfer in plants, making P-containing ashes valuable as fertilizers [109]. While MBA provides a moderate P contribution, it is less effective than high-P ashes such as sewage sludge ash (3.56%) or *M × g* ash from Poland (2.48%). However, compared to biomass fly ash (0.49%) or *Miscanthus sinensis* ash (0.06%), it contains elevated P concentrations and may benefit P-deficient soils. P-containing biomass ashes can be used to increase fertility of soil in degraded lands and reclamation projects [13,20,25]. Many studies have shown that adding biomass ash to soil significantly increases soil P content [20,26,53].

The Si content of MBA in this study was 12.43 ± 1.14%, classifying it as moderate compared to other biomass ashes. It exceeds the Si content of biomass fly ash from residual forest biomass (8.13%) and mixed biomass ash (6.65%) but is lower than that of wood ash (25.4%) and *Miscanthus sinensis* ash (26%). No data were available for *M × g* ash from Poland, sorghum ash, or sewage sludge ash. These findings suggest that MBA has a moderate to lower level of Si compared to other ashes. Si has an important role in plant structural integrity, improving resistance to pests and diseases while increasing tolerance to abiotic stress [110]. The moderate Si content in MBA suggests its potential usefulness in Si-deficient soils, particularly for crops such as rice, sugarcane, and wheat, which require high levels of Si for optimal growth. Compared to biomass fly ash and mixed biomass ash, MBA provides a greater Si contribution, making it a more effective Si source for agricultural applications. However, it remains less effective than wood ash or *Miscanthus sinensis* ash, which contain significantly higher Si concentrations. Moreover, Si-containing ashes can be used for soil stabilization, erosion control, and pozzolanic reactions in construction materials [111]. While an MBA could be utilized in these applications, its effectiveness would be lower than that of high-Si ashes. The moderate Si content in MBA poses minimal environmental risks, as Si is generally non-toxic and exhibits low leaching potential.

The As content of MBA in this study was 8.3 ± 0.42 mg·kg^−1^, classifying it as moderate to high compared to other biomass ashes. It exceeds the As levels reported for *M × g* ash from Poland (0.31 mg·kg^−1^), wood ash (1.82 mg·kg^−1^), sewage sludge ash (1.03 mg·kg^−1^), and *Miscanthus sinensis* ash (5 mg·kg^−1^), but remains lower than biomass fly ash from residual forest biomass (23.5 mg·kg^−1^) and significantly higher than mixed biomass ash, where As levels were below the detection limit (<LOD). As content could be considered as one of the factors in determining the suitability of biomass ash for agricultural and environmental applications [112].

The Cu content of MBA in this study was 33.5 ± 0.71 mg·kg^−1^, classifying it as low to moderate compared to other biomass ashes. It exceeds the Cu levels in sorghum ash (29.7 mg·kg^−1^) and *Miscanthus sinensis* ash (33 mg·kg^−1^) but is substantially lower than biomass fly ash from residual forest biomass (950 mg·kg^−1^), mixed biomass ash (208.7 mg·kg^−1^), sewage sludge ash (311 mg·kg^−1^), *M × g* ash from Poland (70.7 mg·kg^−1^), and wood ash (60.4 mg·kg^−1^). The significantly higher Cu concentrations in sewage sludge ash and biomass fly ash suggest that their feedstocks accumulated more Cu, likely due to industrial or municipal waste inputs, whereas agricultural biomass ashes, including MBA, generally contain lower Cu levels. Cu is an essential micronutrient involved in plant metabolism, enzyme function, and photosynthesis [113,114]. The moderate Cu content in MBA suggests that it can serve as a supplementary Cu source in agricultural soils, particularly in Cu-deficient regions. While its Cu levels are considerably lower than those in sewage sludge ash and biomass fly ash, it provides a higher Cu contribution than some agricultural biomass ashes, such as sorghum ash, making it suitable for controlled use to increase soil micronutrient availability.

The Mn content of MBA in this study was 297 ± 18 mg·kg^−1^, which is relatively low compared to most other biomass ashes. It exceeds the Mn levels in *Miscanthus sinensis* ash (100 mg·kg^−1^) and sorghum ash (148 mg·kg^−1^) but is substantially lower than biomass fly ash from residual forest biomass (7200 mg·kg^−1^), wood ash (7430 mg·kg^−1^), mixed biomass ash (1310 mg·kg^−1^), *M × g* ash from Poland (625.33 mg·kg^−1^), and sewage sludge ash (472 mg·kg^−1^). The significantly higher Mn concentrations in wood ash and biomass fly ash suggest that these feedstocks accumulate and retain more Mn during combustion, whereas agricultural biomass ashes, including MBA, generally contain lower Mn levels. Mn is an essential micronutrient required for plant growth, enzyme activation, and photosynthesis, playing a critical role in chlorophyll synthesis and redox reactions [115]. The moderate Mn content in MBA indicates its capacity as a supplementary Mn source for agricultural use, especially in Mn-deficient soils where availability is restricted due to high pH or soil texture limitations. Compared with sorghum ash and *Miscanthus sinensis* ash, MBA provides a higher Mn contribution, making it a suitable option for soil enrichment when additional Mn input is required. However, it is significantly less effective than wood ash or biomass fly ash as a Mn source. In environmental applications, Mn-containing ashes can be utilized for soil restoration, metal stabilization, and microbial enhancement, as Mn plays a role in oxidation-reduction reactions that influence metal mobility [116]. Given its moderate Mn content, MBA may support these processes, albeit less effectively than high Mn biomass ashes such as wood ash or biomass fly ash. Unlike high-Mn ashes, MBA has a lower risk of excessive Mn accumulation, making it safer for long-term application in most soils. However, in Mn-poor soils, its contribution would be limited, necessitating supplementation with other Mn-rich sources in highly depleted agricultural systems.

The Zn content of MBA in this study was 240.67 ± 49.65 mg·kg^−1^, classifying it as moderate compared to other biomass ashes. It exceeds the Zn content of *M × g* ash from Poland (222.00 mg·kg^−1^) but is lower than that of wood ash (340 mg·kg^−1^), *Miscanthus sinensis* ash (442 mg·kg^−1^), biomass fly ash from residual forest biomass (590 mg·kg^−1^), mixed biomass ash (628.1 mg·kg^−1^), sewage sludge ash (773 mg·kg^−1^), and sorghum ash (1148 mg·kg^−1^). Compared to sewage sludge ash and sorghum ash, which contain significantly higher Zn concentrations, *Miscanthus sinensis* BA retains a lower proportion of Zn from its biomass feedstock, indicating moderate Zn retention during combustion. Zn is an essential plant micronutrient involved in enzyme activation, protein synthesis, and stress tolerance [117,118]. The moderate Zn content in MBA suggests that it can act as a beneficial Zn source in agricultural settings, particularly in Zn-deficient soils. However, it is less effective than high-Zn biomass ashes, such as sewage sludge ash and sorghum ash. Compared with wood ash (340 mg·kg^−1^) and *Miscanthus sinensis* ash (442 mg·kg^−1^), MBA provides a lower Zn contribution, necessitating higher application rates or additional supplementation to achieve comparable effects on soil Zn availability. The moderate Zn content in MBA poses a low risk of Zn toxicity, unlike high-Zn biomass ashes such as sewage sludge ash and sorghum ash, which require stricter application controls. However, repeated applications could cause Zn accumulation, particularly in low-pH soils, where Zn becomes more bioavailable and potentially toxic to plants and soil microorganisms [119]. Careful monitoring of soil conditions and dosage levels is necessary to optimize Zn supplementation and avoid adverse environmental effects.

### 3.2. Potential Contaminants and Regulatory Compliance of MBA

#### 3.2.1. Potentially Toxic Elements (PTEs)

The concentrations of PTEs in MBA were compared with international regulatory standards for the application of biomass ash in soils (Table 4).

The As content of MBA in this study (8.3 mg·kg^−1^) is well below the regulatory limits established by various international standards for its application in soils. Sweden and Lithuania set a maximum permissible As concentration of 30 mg·kg^−1^, while Germany and Finland allow up to 40 mg·kg^−1^. Austria imposes a stricter threshold of 20 mg·kg^−1^ for Class A and B ashes, and Canada enforces the most stringent limit at 14 mg·kg^−1^. Since the As concentration in MBA remains significantly below all these regulatory thresholds, it meets the safety criteria for land application across different regulatory areas.

The Cu content of MBA in this study (33.5 mg·kg^−1^) is also well below international regulatory limits. Sweden and Lithuania permit up to 400 mg·kg^−1^, Finland allows up to 700 mg·kg^−1^, and Austria sets limits of 200 mg·kg^−1^ for Class A ash and 250 mg·kg^−1^ for Class B ash. In Germany, the threshold is stricter at 70 mg·kg^−1^, while France and Canada both impose a maximum allowable concentration of 100 mg·kg^−1^. With a Cu concentration of 33.5 mg·kg^−1^, MBA falls well below even the most restrictive German standard of 70 mg·kg^−1^, confirming its suitability for soil use across all these regulatory frameworks.

The Zn content of MBA in this study (240.67 mg·kg^−1^) falls within the permissible limits established by international standards for biomass ash application in soils. Sweden sets a maximum allowable Zn concentration of 7000 mg·kg^−1^, while Finland permits up to 4500 mg·kg^−1^. Lithuania imposes a threshold of 700 mg·kg^−1^, whereas Austria enforces limits of 1200 mg·kg^−1^ for Class A ash and 1500 mg·kg^−1^ for Class B ash. France restricts Zn content to 300 mg·kg^−1^, while Canada applies the most stringent limit at 220 mg·kg^−1^. With a Zn concentration of 240.67 mg·kg^−1^, MBA complies with all international standards except for Canada’s, where it slightly exceeds the 220 mg·kg^−1^ threshold. However, it remains well below Zn limits in most other countries, confirming its suitability for agricultural and environmental applications.

The concentrations of Cd, Cr, Ni, and Pb in MBA were all below the detection limit (LOD), indicating that these PTEs are either absent or present at concentrations too low to be quantified. This outcome is highly favorable when compared with international regulatory standards for biomass ash application as a soil amendment. The low PTE concentrations detected in the MBA can be primarily attributed to the phytostabilization characteristics of *M × g* [129]. The biomass utilized for combustion originated from several fields surrounding the former Metaleurop Nord Pb smelter in Northern France [130], representing a gradient of soil contamination levels. However, only the aboveground biomass was harvested and used for energy production. Numerous studies have demonstrated that *M × g* behaves predominantly as a phytoexcluder species, exhibiting limited translocation of PTEs from roots to the aboveground parts. In particular, investigations conducted at the Metaleurop Nord site demonstrated that most Cd, Pba, and Zn, along with other PTEs, remain sequestered into the root system and rhizosphere, while concentrations in the harvested stems and leaves remain comparatively low [130,131]. Furthermore, the biomass is harvested at a late stage of the growing cycle (February–March), allowing nutrient remobilization and the translocation of a proportion of accumulated elements from aboveground tissues to belowground organs before harvest. Consequently, the feedstock entering the combustion process already contains relatively low concentrations of PTEs, which largely explains the low concentrations subsequently detected in the resulting ash. Since the MBA in the current study contains Cd, Cr, Ni, and Pb at undetectable levels, it falls well within even the most restrictive international standards, confirming its suitability for agricultural and environmental applications. The absence of detectable concentrations of these PTEs minimizes the risk of soil contamination, plant bioaccumulation, and potential entry into the food chain, further supporting their safe use as soil amendments.

**Figure 2:**
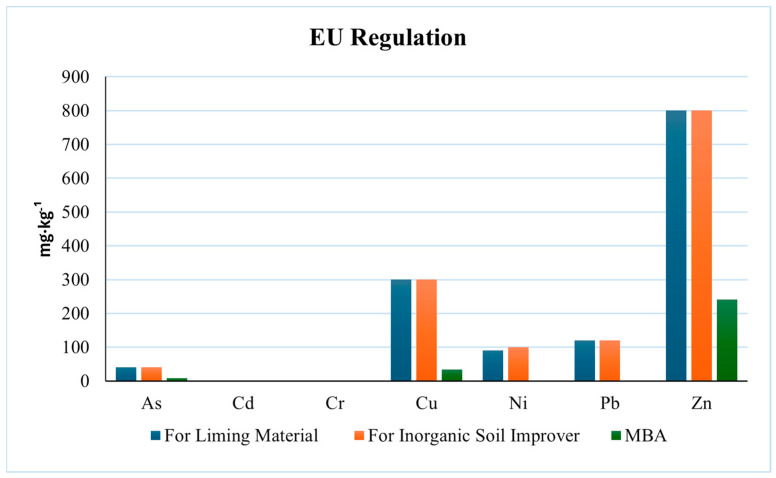
Comparison of PTEs concentrations in the studied MBA with EU regulatory limits for liming materials and inorganic soil improvers.

Although the EU has no ash-specific limits for PTEs in biomass ash used as fertilizer, Regulation (EU) 2019/1009 sets thresholds for products placed on the market as soil amendments [128]. For liming materials, the maxima (mg·kg^−1^, dry matter) are: Cd 2, Cr(VI) 2, Ni 90, Pb 120, As 40, Cu 300, and Zn 800. For inorganic soil improvers, they are: Cd 1.5, Cr(VI) 2, Ni 100, Pb 120, As 40, Cu 300, and Zn 800. MBA contains As 8.3 mg·kg^−1^, Cu 33.5 mg·kg^−1^, and Zn 240.67 mg·kg^−1^, each well below the relevant limits (Figure 2). Cd, Ni, and Pb were not detected; thus, compliance holds provided the analytical LODs are below the corresponding thresholds (Cd 2/1.5; Ni 90/100; Pb 120 mg·kg^−1^). Cr was measured only as total Cr <LOD; because the regulation applies to Cr(VI), confirmation would require Cr(VI) speciation, though an exceedance is unlikely given total Cr is non-detect.

#### 3.2.2. Content of PAHs in MBA

Table 5 presents the concentrations of 16 PAHs in MBA and compares them with those reported in the literature for various biomass ashes to highlight differences in PAH accumulation. Therefore, the PAH concentrations measured in MBA were compared with values reported for other biomass ashes in the literature to evaluate their relative contamination risk. The total PAH content (ΣPAHs) in MBA is 0.66 mg·kg^−1^ (non-detects treated as LOD/2; upper bound < 1.32 mg·kg^−1^). It exceeds values in wood ash (0.01 mg·kg^−1^), coal ash (0.01 mg·kg^−1^), wood-chip ash (0.09 mg·kg^−1^), and mixed biomass ash from coconut and chicken waste (0.311 mg·kg^−1^), but is far lower than wheat-straw fly ash (160 mg·kg^−1^) and wood ash from African traditional cookstoves (10.91 mg·kg^−1^). This pattern indicates more complete combustion than the high-PAH ashes, though less complete than coal and wood-chip systems. For individual PAHs, MBA values are mostly <LOD; using LOD/2, they are generally higher than those in coal and low-PAH wood ash, yet orders of magnitude below wheat-straw fly ash and cookstove wood ash. For example, naphthalene in MBA is <0.02 mg·kg^−1^ (≈0.01 mg·kg^−1^ with LOD/2) versus 0.0001 mg·kg^−1^ in wood ash and 19.1 mg·kg^−1^ in wheat-straw fly ash. Given this low ΣPAHs, MBA presents a low contamination risk relative to high PAH ashes and is suitable for use as a soil conditioner, nutrient source, or liming agent when applied at agronomic rates.

#### 3.2.3. Environmental Risk Assessment of MBA Utilization

The use of MBA in agricultural and environmental applications poses potential risks, though its composition suggests a generally lower risk than that of other biomass ashes. In this study, the risk evaluation should be interpreted as a preliminary assessment based on total PTE concentrations, PAHs, ash alkalinity, salinity, and nutrient composition. Therefore, the risks discussed below represent potential concerns inferred from ash properties rather than effects measured under specific soil types or application rates. Further studies using defined application rates, soil conditions, and incubation or field experiments are needed to quantify these risks more accurately.

PTE contamination is minimal, as Cd, Cr, Ni, and Pb were all below detection limits. Based on the results presented in Section 3.2.1, this significantly reduces concerns regarding soil accumulation, plant uptake, and potential entry into the food chain. However, long-term application should be monitored to prevent PTEs accumulation over time. Soil alkalization remains a potential concern due to the high pH (11.03) of MBA; however, the magnitude of this effect would depend on the ash application rate, soil buffering capacity, initial soil pH, and soil texture. Using high doses could result in over-alkalization, particularly in neutral or slightly alkaline soils, decreasing the availability of essential micronutrients such as Fe, Mn, and P. Soil resistance to pH fluctuations should be considered to prevent pH-induced imbalances of nutrients that may have adverse effects on microbial activity and plant growth. Salt accumulation and soil salinity pose a lesser risk, given the relatively low Na content (0.12%), which is significantly lower than in high Na biomass ashes. However, the presence of K (5.54%) and other soluble ions can still result in localized rises in soil EC if large quantities are applied through repeated use. Regular assessment of soil salinity remains necessary, notably in arid or semi-arid regions where leaching potential is limited [133]. Altered nutrient balance is another potential risk, as the high Ca-to-Mg ratio and elevated levels of K may disrupt CEC and nutrient uptake. An imbalance between K, Ca, and Mg could have a detrimental influence on plant nutrient balance, especially in soils already rich in K or Ca. Long-term application needs to be properly controlled to sustain optimal nutrient ratios and avoid competitive inhibition of essential plant nutrients.

Changes in soil pH and nutrient levels due to MBA may influence soil microbial communities. The high alkalinity and the presence of PTEs, although within safe limits, could have a detrimental effect on microbial diversity and enzyme activity, potentially favoring alkaliphilic microbial populations while suppressing acidophilic and neutrophilic microbes. Such shifts in microbial composition may alter decomposition rates of organic matter and nutrient cycling efficiency, impacting overall soil function. Leaching and groundwater contamination are additional concerns, particularly for P and PTEs such as As and Zn. While the As content of MBA remains well below international regulatory limits, prolonged application in highly permeable or acidic soils could increase its mobility, posing a potential risk to groundwater quality. Similarly, the Zn concentration is within acceptable ranges, but careful monitoring is necessary to control potential overaccumulation in soils with limited cation exchange capacity, where Zn mobility may increase. Although MBA presents a generally low-risk profile for agricultural and environmental applications, careful management is necessary to reduce the possible hazards related to soil alkalization, nutrient imbalances, and PTE leaching. Regular soil monitoring and site-specific application techniques will be essential to ensuring sustainable and safe utilization of MBA.

## 4. Conclusions

This study provides useful evidence for the safe and sustainable valorization of MBA generated from biomass cultivated on contaminated land, within bioenergy waste valorization and circular economy approaches. The characterization of MBA showed a favorable nutrient profile, strong alkalinity, low salinity risk, and low contamination risk based on measured total concentrations of PTEs and PAHs. These properties indicate that MBA can be considered a promising soil amendment, particularly where alkalinity and nutrient supply are beneficial. Despite having these advantages, controlled application remains necessary because excessive ash addition may alter soil pH, increase salinity, disturb nutrient balance, affect microbial activity, and influence plant growth, while contaminant mobility should still be monitored under sensitive soil conditions. Therefore, controlled application rates, periodic soil monitoring, and evaluation under different soil conditions are essential to maximize its benefits while managing associated risks. A limitation of this study is that mineralogical characterization was not conducted; therefore, future studies should incorporate complementary mineralogical and spectroscopic analyses to better clarify the solid-phase composition of MBA and the mechanisms governing nutrient and contaminant mobility in soil–ash systems. In addition, further research should also include leaching toxicity and bioavailability assessments to evaluate contaminant mobility, long-term availability, interactions with soil biogeochemistry and long-term environmental effects under different soil conditions, thereby supporting the safe and effective utilization of MBA in sustainable land management practices.

## Figures and Tables

**Table 1:** CEC, WC, VM, ash content, and FC of MBA, compared with various biomass ashes from the literature [29].

Ash Type	CEC (cmol kg^−1^)	WC (%)	VM (Dry) (%)	Ash (Dry) (%)	FC (Dry) (%)	References
MBA	1.77 ± 0.36	0.47 ± 0.02	3.92 ± 0.44	94.23 ± 0.27	1.85 ± 0.18	This study
High-carbon wood ash	ng	ng	19.63	26.15	ng	Williams & Thomas [39]
Sago Bark Ash	13.13	ng	ng	ng	ng	Hamidi et al. [40]
Wood ash	59.8	ng	20	70.4	9.6	Manirakiza et al. [41]
Mix biomass ash ^a^	ng	ng	1.17	98.67	0.11	Wu et al. [42]
Rice husk ash	40.0	ng	ng	96.0	ng	Severo et al. [43]
Rice mill ash	17.64	ng	ng	ng	ng	Alvarez-Campos et al. [44]

Notes: ng—not given; FC—Fixed C; VM—Volatile matter; WC—water content; ^a^—agricultural and woody biomass.

**Table 2:** Elemental composition (C, N, H, S, and O) of MBA compared with various biomass ashes from the literature.

Ash Type	C (%)	N (%)	H (%)	S (%)	O (%)	References
MBA	5.34	0.02	0.14	0.19	94.41	This study
Wood pellet ash	69.2	0.15	1.4	0.10	24.9	Park et al. [50]
Wood ash	3.25	<LOD	ng	1.72	ng	Mäkinen et al. [51]
*M × g* ash (Croatia)	3.39	0.005	ng	ng	ng	Bhandari et al. [52]
Mix biomass ash ^a^	2.99	0.001	ng	0.47	ng	Szostek et al. [53]
Sorghum ash	3.42	0.50	ng	ng	ng	Romanowska-Duda et al. [26]
Jerusalem artichoke ash	3.40	0.40	ng	ng	ng	
Wood fly ash	0.29	0.05	ng	ng	ng	Ondrasek et al. [54]
Rice straw and husk ash	7.9	<LOD	ng	1.8	46.4	Wu et al. [55]
Amazonian biomass ash ^b^	0.30	0.17	ng	0.12	ng	Albuquerque et al. [56]
Sewage sludge ash	<0.10	<0.08	ng	0.036	ng	Heiskanen et al. [57]
Buckwheat husk ash	29.53	0.66	ng	ng	ng	Pocienė & Šlinkšienė [58]
Rice husk ash	0.93	< 0.01	ng	ng	26.5	Severo et al. [43]
Coal fly ash	1.9	0.04	ng	0.5	ng	Ukwattage et al. [59]
wheat straw fly ash	20.9	0.46	0.62	2.73	6.83	Košnář et al. [60]
Bagasse ash	10.4	0.41	ng	0.05	ng	Benbi et al. [61]

Note: ng—not given; ^a^: (70% forest and 30% agricultural biomass); ^b^: mixture of residual agricultural and forest biomasses.

**Table 3:** Chemical composition and pH of MBA compared with various biomass ashes are presented in the reviewed literature.

Elements	MBA	Biomass Fly Ash ^a^	Mix Biomass Ash ^b^	Sorghum Ash	Sewage Sludge Ash	*M × g* Ash (Poland)	Wood Ash	*Miscanthus sinensis* Ash
pH	11.03 ± 0.04	13.0	12.39	12.0	ng	ng	12.7	ng
Al (%)	0.12 ± 0.01	2.81	0.05	ng	0.89	ng	1.24	0.1
Ca (%)	2.07 ± 0.13	23.9	5.99	3.84	1.76	4.65	13.5	3
Fe (%)	0.24 ± 0.32	2.68	0.22	0.23	0.02	0.25	0.67	ng
K (%)	5.54 ± 0.14	6.13	5.31	16.3	0.21	8.74	3.94	2
Mg (%)	0.37 ± 0.02	2.16	0.86	0.99	0.39	ng	1.27	0.12
Na (%)	0.12 ± 0.01	2.18	0.77	ng	0.21	ng	1.05	0.07
P (%)	0.86 ± 0.08	0.49	1.47	1.42	3.56	2.48	1.0	0.06
Si (%)	12.43 ± 1.14	8.13	6.65	ng	ng	ng	25.4	26
As (mg·kg^−1^)	8.3 ± 0.42	23.5	<LOD	ng	1.03	0.31	1.82	5
Cu (mg·kg^−1^)	33.5 ± 0.71	950	208.7	29.7	311	70.70	60.4	33
Mn (mg·kg^−1^)	297 ± 18	7200	1310	148	472	625.33	7430	100
Zn (mg·kg^−1^)	240.67 ± 49.65	590	628.1	1148	773	222.00	340	442
References	This study	Cruz et al. [78]	Uysal & Yıldızbaş [79]	Romanowska-Duda et al. [26]	Heiskanen et al. [57]	Zając et al. [80]	Maresc et al. [81]	Vigliaturo et al. [82]

Note: ng—not given; ^a^ (residual forest biomass); ^b^ (agricultural and animal wastes).

**Table 4:** Concentration limits of PTEs (mg·kg^−1^) for biomass ash application as a soil amendment [29].

Ash Type	PTEs							References
	As	Cd	Cr	Cu	Ni	Pb	Zn	
MBA	8.3	<LOD	<LOD	33.5	<LOD	<LOD	240.67	This study
Standards								
Sweden	30	30	100	400	70	300	7000	Emilsson [120]
Lithuania	30	30	100	400	70	300	700	Stupak et al. [121]
Germany	40	1.5	2	70	80	150	ng	Silva et al. [122]
Finland	40	25	300	700	150	150	4500	Nurmesniemi et al. [123]
Denmark	ng	5	100	ng	30	120	ng	Niu & Tan [124]
Austria (Class A/B)	20/20	5/8	150/250	200/250	150/200	100/200	1200/1500	Lanzerstorfer [125]
France	ng	2	150	100	50	100	300	Maltas et al. [126]; Ké & Dihé [127]
Canada	14	1.6	120	100	32	60	220	Ké & Dihé [127]
EU liming materials	40	2	2	300	90	120	800	[128]
EU inorganic soil improvers	40	1.5	2	300	100	120	800	[128]

Note: <LOD = below detection limit; ng: not given.

**Table 5:** Concentrations of 16 PAHs (mg·kg^−1^) in MBA compared with various biomass ashes from the literature [29].

16 PAHs (mg·kg^−1^)	MBA	Wood Ash	Coal Ash	Wood Chips Ash	Wood Ash ^a^	Wheat Straw Fly Ash	Mix Biomass Ash ^b^
Naphthalene	˂0.02	0.0001	<LOD	0.08	0.035	19.1	0.055
Acenaphthylene	<0.05	0.00006	<LOD	ng	0.342	12.6	0.021
Acenaphthene	<0.05	0.0005	0.0001	ng	0.188	3.87	0.009
Fluorene	<0.02	0.001	0.0007	ng	0.189	0.17	0.008
Anthracene	<0.05	0.0002	0.0003	ng	2.925	21.7	0.015
Phenanthrene	<0.05	0.001	0.001	ng	0.280	17.9	0.091
Fluoranthene	<0.02	0.001	0.001	ng	0.581	16.3	0.028
Pyrene	<0.02	0.0002	0.0001	ng	0.719	6.54	0.019
Benz[a]anthracene	<0.1	0.004	0.005	ng	0.479	7.28	<0.0004
Chrysene	<0.1	0.0007	0.0006	ng	0.729	6.49	<0.0003
Benzo[k]fluoranthene	<0.2	0.0002	0.0001	ng	1.277	7.57	<0.001
Benzo[b]fluoranthene	<0.02	0.0003	0.0002	ng	1.275	13.8	0.060
Benzo[a]pyrene	<0.1	0.0002	0.0002	ng	0.396	15.0	0.005
Indeno[1,2,3-cd]pyrene	<0.5	0.0001	0.0001	ng	0.414	1.15	<0.006
Dibenz[a,h]anthracene	<0.2	0.0001	0.00008	ng	0.153	3.87	<0.006
Benzo[g,h,i]perylene	<0.05	0.00004	0.00003	ng	1.026	6.69	<0.006
ΣPAHs	0.66 ^c^	0.01	0.01	0.09	10.91	160	0.311
References	This study	Kozielska et al. [132]	Kozielska et al. [132]	Ondrasek et al. [133]	Etchie et al. [134]	Košnář et al. [60]	Masto et al. [135]

Note: ^a^ (Wood ash from the African traditional cookstoves); ^b^ (coconut and chicken waste); ^c^ Calculated as LOD/2 for non-detects.

## Data Availability

The original contributions presented in this study are included in the article. Further inquiries can be directed to the corresponding author.
